# Supplementary material for: Validation of Variant Assembly Using HAPHPIPE with Next-Generation Sequence Data from Viruses
Source: Viruses. 2020 Jul 14;12(7):758. doi: 10.3390/v12070758 (PMC7412389; doi:10.3390/v12070758)
Supplement: Supplementary file 1 [file viruses-12-00758-s001.pdf]

Validation of variant assembly using HAPHPIPE with next generation sequence data from viruses

Keylie M. Gibson [1,^,*], Margaret C. Steiner [1,^], Uzma Rentia [1], Matthew L. Bendall [1], Marcos Pérez-Losada [1,2,3], and Keith A. Crandall [1,2]

[1] Computational Biology Institute and [2] Department of Biostatistics and Bioinformatics, Milken Institute School of Public Health, The George Washington University, Washington DC 20052, USA
[3] CIBIO-InBIO, Centro de Investigação em Biodiversidade e Recursos Genéticos, Universidade do Porto, Campus Agrário de Vairão, Vairão, Portugal
^ Co-first authors
* Correspondence: kmgibson@gwu.edu

Supplemental Material

Figure S1. Genetic p-distance (displayed as a difference from 1) between consensus sequence and true sequence for all pipelines for the simulated HIV A) subtype B dataset and B) non-subtype B dataset. A value closer to 1.00 indicates the consensus sequence is more genetically similar to the true sequence. The x-axis order from left to right for an individual panel: adjusted genetic p-distance between the true sequence and (i) the initial assembled sequence followed by (ii) the final assemble sequence for haphpipe_assemble_01 pipeline (de novo assembly); (iii) the initial assembled sequence followed by (iv) the final assemble sequence for haphpipe_assemble_02 pipeline (reference-based assembly); the final consensus sequence for the Geneious (v) de novo workflow and the (vi) reference-based workflow; and finally, the (vi) average between the final two sequences (one for each read file) for HyDRA. The three amplicons are shown, as well as a combination of *PRRT* and *int* amplicons into *pol*. There are no results for HyDRA in the *gp120* gene because HyDRA only analyzes the *pol* gene.

Figure S2. Genetic p-distance (displayed as a difference from 1) between consensus sequence and HXB2, the reference sequence for HIV, for all pipelines for the simulated HIV A) subtype B dataset and B) non-subtype B dataset. Ambiguous nucleotides were accounted for by giving fractional weight in alignment. A value closer to 1.00 indicates that the consensus sequence is more genetically similar to the reference sequence. The x-axis order from left to right for an individual panel: adjusted genetic p-distance between the reference sequence and (i) the initial assembled sequence followed by (ii) the final assemble sequence for haphpipe_assemble_01 pipeline (de novo assembly); (iii) the initial assembled sequence followed by (iv) the final assemble sequence for haphpipe_assemble_02 pipeline (reference-based assembly); the final consensus sequence for the Geneious (v) de novo workflow and the (vi) reference-based workflow; and finally, the (vi) average between the final two sequences (one for each read file) for HyDRA. The three amplicons are shown, as well as a combination of *PRRT* and *int* amplicons into *pol*. There are no results for HyDRA in the *gp120* gene because HyDRA only analyzes the *pol* gene.

Figure S3. Genetic p-distance (displayed as a difference from 1) between consensus sequence and HXB2, the reference sequence for HIV, for all pipelines for the empirical A) HIV dataset and B) HCV dataset. Ambiguous nucleotides were accounted for by giving fractional weight in alignment. A value closer to 1.00 indicates that the consensus sequence is more genetically similar to the reference sequence. The y-axes are different for each HIV and HCV, with HCV showing greater variance between samples. The x-axis order from left to right for an individual panel: adjusted genetic p-distance between the reference sequence and (i) the initial assembled sequence, (ii) the final assemble sequence and (iii) the reconstructed haplotypes for haphpipe_assemble_01 pipeline (de novo assembly); (iv) the initial assembled sequence, (v) the final assemble sequence, and (vi) the reconstructed haplotypes for haphpipe_assemble_02 pipeline (reference-based assembly); the final consensus sequence for the Geneious (vii) de novo workflow and (vi) reference-based workflow; and finally, the (viii) average between the final two sequences (one for each read file) for HyDRA. The three amplicons are shown for both empirical datasets (HIV: *PRRT*, *int*, *gp120* and HCV: *core*, *E1*, *E2*). There are no results for HyDRA in the *gp120* gene for HIV or for any HCV genes because HyDRA only analyzes the *pol* gene region of HIV.

Table S1. Accessions used in validation study.

Table S2. Kruskal-Wallis rank-sum test of the genetic p-distance and adjusted genetic p-distance of the pipeline consensus sequence from the true sequence or reference sequence for all datasets. P-values are reported (Holm adjustment).

Table S3. Wilcoxon signed-rank comparisons of the genetic p-distance from the true sequence between the initial and final consensus sequences constructed in the HAPHPIPE pipelines for the simulation dataset.

Table S4. Kruskal-Wallis multiple comparisons (Dunn test) of the genetic p-distance of the pipeline consensus sequence from the true sequence for the simulation dataset. P-values are reported (Holm adjustment).

Table S5. Wilcoxon signed-rank comparisons of the genetic p-distance from HXB2, the HIV reference sequence, between the initial and final consensus sequences constructed in the HAPHPIPE pipelines for the simulation dataset.

Table S6. Kruskal-Wallis multiple comparisons (Dunn test) of the genetic p-distance of the pipeline consensus sequence from HXB2, the HIV reference sequence, for the simulation dataset. P-values are reported (Holm adjustment).

Table S7. Kruskal-Wallis multiple comparisons (Dunn test) of the genetic p-distance of the pipeline consensus sequence from HXB2, the HIV reference sequence, for the HIV empirical dataset. Adjusted p-values are reported (Holm adjustment).

Table S8. Kruskal-Wallis multiple comparisons (Dunn test) of the genetic p-distance of the pipeline consensus sequence from H77, the HCV reference sequence, for the HCV empirical dataset. Adjusted p-values are reported (Holm adjustment).

Table S9. Wilcoxon signed-rank comparisons of the genetic p-distance from the reference sequence between the initial and final consensus sequences constructed in the HAPHPIPE pipelines for the empirical HIV and HCV datasets. The reference sequences for HIV and HCV were HXB2 and H77, respectively.

Table S10. Genetic p-distance to the reference sequence across the empirical SARS-CoV-2 dataset.

Table S1. Accessions used in validation study.

Subtype B	Reference	Non-Subtype B	Reference
AB221125	[1]	AF069673	[2]
AB428552	[3]	AF082394	[4]
AF042103	[5]	AF361872	[6]
AJ271445	[7]	AF443107	[8]
AY173955, AY173960	[9]	AF484477, AF484489, AF484498	[10]
AY781126	[11]	AJ249239	[12]
AY795904, AY795905	[13]	AY253305	[14]
AY835758, AY835763	[15]	AY371155	[16]
DQ127549	[17]	AY563169	[18]
DQ676885	[19]	AY967806	[20]
DQ886035	[21]	DQ093592, DQ275650	[22]
EF514700, EF514711	[23]	DQ676872	[19]
EU839601, EU839603, EU839606	[24]	EF614151	[25]
FJ195086	[26]	FJ623475	[27]
FJ388904, FJ388919	[28]	GQ999977, GQ999982, GQ999988, GQ999991	[29]
FJ469714, FJ469719, FJ469722, FJ469745, FJ469748, FJ469758, FJ469771	[30]	JX140664, JX140672<br>KJ948662	[31]<br>[32]
FJ495941, FJ496000	[33]	KR017776	[34]
FJ853622	[35]	KT022378	[36]
HM586209	[37]	KU319533, KU319539	[38]
JF320018, JF320189, JF320263	[39]	KX232609	[40]
JF683751, JF683793	[41]	KX232610	[42]
JF689856, JF689874, JF689886, JF689893, JF689897	[43]	KX907346, KX907368, KX907389, KX907394	[44]
JF932475, JF932490	[45]	KY392779	[46]
JN248321, JN248353	[47]	MF373128, MF373168	[48]
JN692480	[49]	AB287376, AB485648, AF107771, KC156214, KF716467, KP109483, KP109525, KU749392, KU749422, KY275364, KY496624, KY658694, KY658709	None
JQ403075, JQ403098, JQ403105	[50]		
JX140652, JX140654	[31]		
JX446800	[51]		
JX960598	[52]		
K02007	[53]		
KC899011	[54]		
KF384800	[55]		
KJ140266	[56]		
KJ849801	[57]		
KP411827	[58]		
KP411829	[58]		

KR914678	[59]		
KT124749, KT124756, KT124778, KT124783, KT124796, KT124797	[60]		
KT427675, KT427704, KT427714, KT427719, KT427730, KT427737, KT427744, KT427803	[61]		
KU168260	[62]		
KX505555	[63]		
KY778615	[64]		
KY968395, KY968403	[65]		
L02317	[66]		
MF373129, MF373201	[48]		
U43096	[40]		
U71182	[67]		
AB289590, AB565497, AY560107, DQ322225, JN251896, KC473830, KC473834, KF716498, KP109515, KT276264, KU685591, KU749389, KY658690	None		

1. Leal E, Villanova FE. Diversity of HIV-1 Subtype B: Implications to the Origin of BF Recombinants. PLoS One. 2010;5: e11833. doi:10.1371/ journal.pone.0011833
2. Carr JK, Laukkanen T, Salminen MO, Albert J, Alaeus A, Kim B, et al. Characterization of subtype A HIV-1 from Africa by full genome sequencing. Aids. 1999;13: 1819–1826. doi:10.1097/00002030-199910010-00003
3. Ibe S, Shigemi U, Sawaki K, Fujisaki S, Hattori J, Yokomaku Y, et al. Analysis of near full-length genomic sequences of drug-resistant HIV-1 spreading among therapy-naive individuals in Nagoya, Japan: amino acid mutations associated with viral replication activity. AIDS Res Hum Retroviruses. United States; 2008;24: 1121–1125. doi:10.1089/aid.2008.0090
4. Laukkanen T, Albert J, Liitsola K, Green SD, Carr JK, Leitner T, et al. Virtually full-length sequences of HIV type 1 subtype J reference strains. AIDS Res Hum Retroviruses. United States; 1999;15: 293–297. doi:10.1089/088922299311475
5. Oelrichs R, Tsykin A, Rhodes D, Solomon A, Ellett A, McPhee D, et al. Genomic sequence of HIV type 1 from four members of the Sydney Blood Bank Cohort of long-term nonprogressors. AIDS Res Hum Retroviruses. United States; 1998;14: 811–814. doi:10.1089/aid.1998.14.811
6. Hoelscher M, Kim B, Maboko L, Mhalu F, Von Sonnenburg F, Birx DL, et al. High proportion of unrelated HIV-1 intersubtype recombinants in the Mbeya region of southwest Tanzania. Aids. 2001;15: 1461–1470. doi:10.1097/00002030-200108170-00002
7. Novelli P, Vella C, Oxford J, Daniels RS. Construction and biological characterization of an infectious molecular clone of HIV type 1GB8. AIDS Res Hum Retroviruses. United States; 2000;16: 1175–1178. doi:10.1089/088922200415027
8. Novitsky V, Smith UR, Gilbert P, McLane MF, Chigwedere P, Williamson C, et al. Human Immunodeficiency Virus Type 1 Subtype C Molecular Phylogeny: Consensus Sequence for an AIDS Vaccine Design? J Virol. 2002;76: 5435–5451. doi:10.1128/jvi.76.11.5435-5451.2002
9. Hierholzer J, Montano S, Hoelscher M, Negrete M, Hierholzer M, Avila MM, et al. Molecular Epidemiology of HIV Type 1 in Ecuador, Peru, Bolivia, Uruguay, and Argentina. AIDS Res Hum

Retroviruses. United States; 2002;18: 1339–1350. doi:10.1089/088922202320935410
10. Harris ME, Serwadda D, Sewankambo N, Kim B, Kigozi G, Kiwanuka N, et al. Among 46 near full length HIV type 1 genome sequences from Rakai District, Uganda, subtype D and AD recombinants predominate. AIDS Res Hum Retroviruses. United States; 2002;18: 1281–1290. doi:10.1089/088922202320886325
11. Viñoles J, Serra M, Russi JC, Ruchansky D, Sosa-Estani S, Montano SM, et al. Seroincidence and phylogeny of human immunodeficiency virus infections in a cohort of commercial sex workers in Montevideo, Uruguay. Am J Trop Med Hyg. 2005;72: 495–500. doi:10.4269/ajtmh.2005.72.495
12. Triques K, Bourgeois A, Vidal N, Mpoudi-Ngole E, Mulanga-Kabeya C, Nzilambi N, et al. Near-full-length genome sequencing of divergent African HIV type 1 subtype F viruses leads to the identification of a new HIV type 1 subtype designated K. AIDS Res Hum Retroviruses. United States; 2000;16: 139–151. doi:10.1089/088922200309485
13. Saad MD, Al-Jaufy A, Grahan RR, Nadai Y, Earhart KC, Sanchez JL, et al. HIV type 1 strains common in Europe, Africa, and Asia cocirculate in Yemen. AIDS Res Hum Retroviruses. United States; 2005;21: 644–648. doi:10.1089/aid.2005.21.644
14. Arroyo MA, Hoelscher M, Sanders-Buell E, Herbinger K-H, Samky E, Maboko L, et al. HIV type 1 subtypes among blood donors in the Mbeya region of southwest Tanzania. AIDS Res Hum Retroviruses. United States; 2004;20: 895–901. doi:10.1089/0889222041725235
15. Mikhail M, Wang B, Lemey P, Beckthold B, Vandamme AM, Gill MJ, et al. Role of viral evolutionary rate in HIV-1 disease progression in a linked cohort. Retrovirology. 2005;2: 1–10. doi:10.1186/1742-4690-2-41
16. Kijak GH, Sanders-Buell E, Wolfe ND, Mpoudi-Ngole E, Kim B, Brown B, et al. Development and application of a high-throughput HIV type 1 genotyping assay to identify CRF02_AG in West/West Central Africa. AIDS Res Hum Retroviruses. United States; 2004;20: 521–530. doi:10.1089/088922204323087778
17. Allen TM, Altfeld M, Geer SC, Kalife ET, Moore C, Desouza I, et al. Selective Escape from CD8⩽ T-Cell Responses Represents a Major Driving Force of Human Immunodeficiency Virus Type 1 (HIV-1) Sequence Diversity and Reveals Constraints on HIV-1 Evolution. J Virol. 2005;79: 13239–13249. doi:10.1128/JVI.79.21.13239
18. Carrion G, Eyzaguirre L, Montano SM, Laguna-Torres V, Serra M, Aguayo N, et al. Documentation of subtype C HIV Type 1 strains in Argentina, Paraguay, and Uruguay. AIDS Res Hum Retroviruses. United States; 2004;20: 1022–1025. doi:10.1089/aid.2004.20.1022
19. Li B, Gladden AD, Altfeld M, Kaldor JM, Cooper DA, Kelleher AD, et al. Rapid Reversion of Sequence Polymorphisms Dominates Early Human Immunodeficiency Virus Type 1 Evolution. J Virol. 2007;81: 193–201. doi:10.1128/jvi.01231-06
20. Qiu Z, Xing H, Wei M, Duan Y, Zhao Q, Xu J, et al. Characterization of five nearly full-length genomes of early HIV type 1 strains in Ruili city: implications for the genesis of CRF07_BC and CRF08_BC circulating in China. AIDS Res Hum Retroviruses. United States; 2005;21: 1051–1056. doi:10.1089/aid.2005.21.1051
21. Frahm N, Kaufmann DE, Yusim K, Muldoon M, Kesmir C, Linde CH, et al. Increased Sequence Diversity Coverage Improves Detection of HIV-Specific T Cell Responses. J Immunol. 2007;179: 6638–6650. doi:10.4049/jimmunol.179.10.6638
22. Rousseau CM, Birditt BA, McKay AR, Stoddard JN, Lee TC, McLaughlin S, et al. Large-scale amplification, cloning and sequencing of near full-length HIV-1 subtype C genomes. J Virol Methods. Netherlands; 2006;136: 118–125. doi:10.1016/j.jviromet.2006.04.009
23. Andresen BS, Vinner L, Tang S, Bragstad K, Kronborg G, Gerstoft J, et al. Characterization of near full-length genomes of HIV type 1 strains in Denmark: basis for a universal therapeutic vaccine. AIDS Res Hum Retroviruses. United States; 2007;23: 1442–1448. doi:10.1089/aid.2007.0111

24. Nadai Y, Eyzaguirre LM, Sill A, Cleghorn F, Nolte C, Charurat M, et al. HIV-1 epidemic in the Caribbean is dominated by subtype B. PLoS One. 2009;4: 1–5. doi:10.1371/journal.pone.0004814
25. Abecasis AB, Lemey P, Vidal N, de Oliveira T, Peeters M, Camacho R, et al. Recombination Confounds the Early Evolutionary History of Human Immunodeficiency Virus Type 1: Subtype G Is a Circulating Recombinant Form. J Virol. 2007;81: 8543–8551. doi:10.1128/jvi.00463-07
26. Diaz RS, Leal É, Sanabani S, Sucupira MCA, Tanuri A, Sabino EC, et al. Selective regimes and evolutionary rates of HIV-1 subtype B V3 variants in the Brazilian epidemic. Virology. Elsevier Inc.; 2008;381: 184–193. doi:10.1016/j.virol.2008.08.014
27. Tovanabutra S, Sanders EJ, Graham SM, Mwangome M, Peshu N, McClelland RS, et al. Evaluation of HIV type 1 strains in men having sex with men and in female sex workers in Mombasa, Kenya. AIDS Res Hum Retroviruses. United States; 2010;26: 123–131. doi:10.1089/aid.2009.0115
28. Kousiappa I, Van De Vijver DAMC, Kostrikis LG. Near full-length genetic analysis of HIV sequences derived from Cyprus: evidence of a highly polyphyletic and evolving infection. AIDS Res Hum Retroviruses. United States; 2009;25: 727–740. doi:10.1089/aid.2008.0239
29. Treurnicht FK, Seoighe C, Martin DP, Wood N, Abrahams M-R, Rosa D de A, et al. Adaptive changes in HIV-1 subtype C proteins during early infection are driven by changes in HLA-associated immune pressure. Virology. 2010;396: 213–225. doi:10.1016/j.virol.2009.10.002. Adaptive
30. Wang YE, Li B, Carlson JM, Streeck H, Gladden AD, Goodman R, et al. Protective HLA Class I Alleles That Restrict Acute-Phase CD8+ T-Cell Responses Are Associated with Viral Escape Mutations Located in Highly Conserved Regions of Human Immunodeficiency Virus Type 1. J Virol. 2009;83: 1845–1855. doi:10.1128/jvi.01061-08
31. Hora B, Keating SM, Chen Y, Sanchez AM, Sabino E, Hunt G, et al. Genetic characterization of a panel of diverse HIV-1 isolates at seven international sites. PLoS One. 2016;11: 1–18. doi:10.1371/journal.pone.0157340
32. Wilkinson E, Holzmayer V, Jacobs GB, De Oliveira T, Brennan CA, Hackett J, et al. Sequencing and phylogenetic analysis of near full-length HIV-1 subtypes A, B, G and unique recombinant AC and AD viral strains identified in South Africa. AIDS Res Hum Retroviruses. 2015;31: 412–420. doi:10.1089/aid.2014.0230
33. Salazar-Gonzalez JF, Salazar MG, Keele BF, Learn GH, Giorgi EE, Li H, et al. Genetic identity, biological phenotype, and evolutionary pathways of transmitted/founder viruses in acute and early HIV-1 infection. J Exp Med. 2009;206: 1273–1289. doi:10.1084/jem.20090378
34. Tongo M, Dorfman JR, Abrahams MR, Mpoudi-Ngole E, Burgers WA, Martin DP. Near full-length HIV type 1M genomic sequences from Cameroon. Evol Med Public Heal. 2015;2015: 254–265. doi:10.1093/emph/eov022
35. Cuevas MT, Fernandez-Garcia A, Pinilla M, Garcia-Alvarez V, Thomson M, Delgado E, et al. Short communication: Biological and genetic characterization of HIV type 1 subtype B and nonsubtype B transmitted viruses: usefulness for vaccine candidate assessment. AIDS Res Hum Retroviruses. United States; 2010;26: 1019–1025. doi:10.1089/aid.2010.0018
36. Billings E, Sanders-Buell E, Bose M, Bradfield A, Lei E, Kijak GH, et al. The number and complexity of pure and recombinant HIV-1 strains observed within incident infections during the HIV and malaria cohort study conducted in Kericho, Kenya, from 2003 to 2006. PLoS One. 2015;10: 1–17. doi:10.1371/journal.pone.0135124
37. Turnbull EL, Wong M, Wang S, Wei X, Jones NA, Conrod KE, et al. Kinetics of Expansion of Epitope-Specific T Cell Responses during Primary HIV-1 Infection. J Immunol. 2009;182: 7131–7145. doi:10.4049/jimmunol.0803658
38. Amogne W, Bontell I, Grossmann S, Aderaye G, Lindquist L, Sonnerborg A, et al. Phylogenetic Analysis of Ethiopian HIV-1 Subtype C Near Full-Length Genomes Reveals High Intrasubtype Diversity and a Strong Geographical Cluster. AIDS Res Hum Retroviruses. United States; 2016;32:

471–474. doi:10.1089/aid.2015.0380
39. Rolland M, Tovanabutra S, Allan C, Frahm N, Peter B, Sanders-buell E, et al. Genetic impact of vaccination on breakthrough HIV-1 sequences from the Step trial. Nat Med. 2011;17: 366–371. doi:10.1038/nm.2316.Genetic
40. Kreutz R, Dietrich U, Kuhnel H, Nieselt-Struwe K, Eigen M, Rubsamen-Waigmann H. Analysis of the envelope region of the highly divergent HIV-2ALT isolate extends the known range of variability within the primate immunodeficiency viruses. AIDS Res Hum Retroviruses. United States; 1992;8: 1619–1629. doi:10.1089/aid.1992.8.1619
41. Kousiappa I, Achilleos C, Hezka J, Lazarou Y, Othonos K, Demetriades I, et al. Molecular characterization of HIV type 1 strains from newly diagnosed patients in Cyprus (2007-2009) recovers multiple clades including unique recombinant strains and lack of transmitted drug resistance. AIDS Res Hum Retroviruses. United States; 2011;27: 1183–1199. doi:10.1089/aid.2011.0060
42. Chen Y, Hora B, DeMarco T, Shah SA, Ahmed M, Sanchez AM, et al. Fast dissemination of new HIV-1 CRF02/A1 recombinants in Pakistan. PLoS One. 2016;11. doi:10.1371/journal.pone.0167839
43. Eyzaguirrea LM, Charurata M, Redfielda RR, Blattnera WA, Carra JK, Sajadi MM. Elevated hypermutation levels in HIV-1 natural viral suppressors. Virology. 2013;443: 306–312. doi:10.1016/j.virol.2013.05.019
44. Billings E, Sanders-Buell E, Bose M, Kijak GH, Bradfield A, Crossler J, et al. HIV-1 Genetic Diversity Among Incident Infections in Mbeya, Tanzania. AIDS Res Hum Retroviruses. 2017;33: 373–381. doi:10.1089/AID.2016.0111
45. Li Z, He X, Wang Z, Xing H, Li F, Yang Y, et al. Tracing the origin and history of HIV-1 subtype B′ epidemic by near full-length genome analyses. Aids. 2012;26: 877–884. doi:10.1097/QAD.0b013e328351430d
46. Rodgers MA, Wilkinson E, Vallari A, McArthur C, Sthreshley L, Brennan CA, et al. Sensitive Next-Generation Sequencing Method Reveals Deep Genetic Diversity of HIV-1 in the Democratic Republic of the Congo. J Virol. 2017;91: 1–18. doi:10.1128/jvi.01841-16
47. Kijak GH, Tovanabutra S, Rerks-Ngarm S, Nitayaphan S, Eamsila C, Kunasol P, et al. Molecular Evolution of the HIV-1 Thai Epidemic between the Time of RV144 Immunogen Selection to the Execution of the Vaccine Efficacy Trial. J Virol. 2013;87: 7265–7281. doi:10.1128/jvi.03070-12
48. Neogi U, Siddik AB, Kalaghatgi P, Gisslén M, Bratt G, Marrone G, et al. Recent increased identification and transmission of HIV-1 unique recombinant forms in Sweden. Sci Rep. 2017;7: 1–9. doi:10.1038/s41598-017-06860-2
49. Sanabani SS, de Pastena ÉRS, da Costa AC, Martinez VP, Kleine-Neto W, de Oliveira ACS, et al. Characterization of partial and near full-length genomes of HIV-1 strains sampled from recently infected individuals in São Paulo, Brazil. PLoS One. 2011;6: 1–11. doi:10.1371/journal.pone.0025869
50. Henn MR, Boutwell CL, Charlebois P, Lennon NJ, Power KA, Macalalad AR, et al. Whole genome deep sequencing of HIV-1 reveals the impact of early minor variants upon immune recognition during acute infection. PLoS Pathog. 2012;8: e1002529. doi:10.1371/journal.ppat.1002529
51. Edlefsen PT, Rolland M, Hertz T, Tovanabutra S, Gartland AJ, deCamp AC, et al. Comprehensive Sieve Analysis of Breakthrough HIV-1 Sequences in the RV144 Vaccine Efficacy Trial. PLoS Comput Biol. 2015;11: 1–37. doi:10.1371/journal.pcbi.1003973
52. An M, Han X, Xu J, Chu Z, Jia M, Wu H, et al. Reconstituting the Epidemic History of HIV Strain CRF01_AE among Men Who Have Sex with Men (MSM) in Liaoning, Northeastern China: Implications for the Expanding Epidemic among MSM in China. J Virol. 2012;86: 12402–12406. doi:10.1128/jvi.00262-12
53. Sanchez-Pescador R, Power MD, Barr PJ, Steimer KS, Stempien MM, Brown-Shimer SL, et al. Nucleotide sequence and expression of an AIDS-associated retrovirus (ARV-2). Science (80-).

United States; 1985;227: 484–492. doi:10.1126/science.2578227

54. Han X, An M, Zhao B, Duan S, Yang S, Xu J, et al. High Prevalence of HIV-1 Intersubtype B′/C Recombinants among Injecting Drug Users in Dehong, China. PLoS One. 2013;8. doi:10.1371/journal.pone.0065337
55. Salgado M, Swanson MD, Pohlmeyer CW, Buckheit RW, Wu J, Archin NM, et al. HLA-B*57 Elite Suppressor and Chronic Progressor HIV-1 Isolates Replicate Vigorously and Cause CD4+ T Cell Depletion in Humanized BLT Mice. J Virol. 2014;88: 3340–3352. doi:10.1128/jvi.03380-13
56. Cho YK, Kim JE, Foley BT. Phylogenetic analysis of near full-length HIV type 1 genomic sequences from 21 korean individuals. AIDS Res Hum Retroviruses. 2013;29: 738–743. doi:10.1089/aid.2012.0298
57. Pessôa R, Watanabe JT, Calabria P, Alencar CS, Loureiro P, Lopes ME, et al. Enhanced detection of viral diversity using partial and near full- length genomes of HIV-1 provirus deep sequencing data from recently infected donors at four blood centers in Brazil. Transfusion. 2015;55: 980–990. doi:10.1111/trf.12936.Enhanced
58. Grossmann S, Nowak P, Neogi U. Subtype-independent near full-length HIV-1 genome sequencing and assembly to be used in large molecular epidemiological studies and clinical management. J Int AIDS Soc. 2015;18: 1–8. doi:10.7448/IAS.18.1.20035
59. Blanco M, Machado LY, Díaz H, Ruiz N, Romay D, Silva E. HIV-1 genetic variability in Cuba and implications for transmission and clinical progression. MEDICC Rev. 2015;17: 25–31.
60. Tully DC, Ogilvie CB, Batorsky RE, Bean DJ, Power KA, Ghebremichael M, et al. Differences in the Selection Bottleneck between Modes of Sexual Transmission Influence the Genetic Composition of the HIV-1 Founder Virus. PLoS Pathog. 2016;12: 1–29. doi:10.1371/journal.ppat.1005619
61. Pessôa R, Loureiro P, Lopes ME, Carneiro-Proietti ABF, Sabino EC, Busch MP, et al. Ultra-deep sequencing of HIV-1 near full-length and partial proviral genomes reveals high genetic diversity among Brazilian blood donors. PLoS One. 2016;11: 1–14. doi:10.1371/journal.pone.0152499
62. Berg MG, Yamaguchi J, Alessandri-Gradt E, Tell RW, Plantier JC, Brennan CA. A pan-HIV strategy for complete genome sequencing. J Clin Microbiol. 2015/12/25. 2016;54: 868–882. doi:10.1128/JCM.02479-15
63. Bruner KM, Murray AJ, Pollack RA, Soliman MG, Sarah B, Capoferri AA, et al. Defective proviruses rapidly accumulate during acute HIV-1 infection Katherine. Nat Med. 2016;22: 1043–1049. doi:10.1038/nm.4156.Defective
64. Hiener B, Eden J, Horsburgh BA, Palmer S. Amplification of Near Full-length HIV-1 Proviruses for Next-Generation Sequencing. J Vis Exp. 2018;140: e58016. doi:10.3791/58016
65. Cevallos CG, Jones LR, Pando MA, Carr JK, Avila MM, Quarleri J. Genomic characterization and molecular evolution analysis of subtype B and BF recombinant HIV-1 strains among Argentinean men who have sex with men reveal a complex scenario. PLoS One. 2017;12: 1–12. doi:10.1371/journal.pone.0189705
66. Ghosh SK, Fultz PN, Keddie E, Saag MS, Sharp PM, Hahn BH, et al. A Molecular Clone of HIV-1 Tropic and Cytopathic for Human and Chimpanzee Lymphocytes. Virology. 1993. pp. 858–864. doi:10.1006/viro.1993.1331
67. Graf M, Shao Y, Zhao Q, Seidl T, Kostler J, Wolf H, et al. Cloning and characterization of a virtually full-length HIV type 1 genome from a subtype B′-Thai strain representing the most prevalent B-clade isolate in China. AIDS Res Hum Retroviruses. United States; 1998;14: 285–288. doi:10.1089/aid.1998.14.285

Table S2. Kruskal-Wallis rank-sum test of the genetic p-distance and adjusted genetic p-distance of the pipeline consensus sequence from the true sequence or reference sequence for all datasets. P-values are reported (Holm adjustment).

**Simulation HIV Subtype B dataset from true sequence**			**Simulation HIV Non-subtype B dataset from true sequence**		
**Gene**	**PDIST**	**APDIST**	**Gene**	**PDIST**	**APDIST**
pol	< 2.2e-16***	< 2.2e-16***	***pol***	< 2.2e-16***	< 2.2e-16***
PRRT	< 2.2e-16***	< 2.2e-16***	***PRRT***	< 2.2e-16***	< 2.2e-16***
int	< 2.2e-16***	< 2.2e-16***	***int***	< 2.2e-16***	< 2.2e-16***
gp120	< 2.2e-16***	< 2.2e-16***	***gp120***	< 2.2e-16***	< 2.2e-16***
**Simulation HIV Subtype B dataset from HXB2 reference sequence**			**Simulation HIV Non-subtype B dataset from HXB2 reference sequence**		
**Gene**	**PDIST**	**APDIST**	**Gene**	**PDIST**	**APDIST**
pol	< 2.2e-16***	< 2.2e-16***	***pol***	< 2.2e-16***	< 2.2e-16***
PRRT	< 2.2e-16***	< 2.2e-16***	***PRRT***	< 2.2e-16***	7.8E-14***
int	< 2.2e-16***	< 2.2e-16***	***int***	< 2.2e-16***	< 2.2e-16***
gp120	9.6E-11***	0.0799	***gp120***	< 2.2e-16***	< 2.2e-16***
**Empirical HIV dataset from HXB2 reference sequence**			**Empirical HCV dataset from H77 reference sequence**		
**Gene**	**PDIST**	**APDIST**	**Gene**	**PDIST**	**APDIST**
PRRT	< 2.2e-16***	< 2.2e-16***	***core***	< 2.2e-16***	< 2.2e-16***
int	< 2.2e-16***	< 2.2e-16***	***E1***	< 2.2e-16***	< 2.2e-16***
gp120	< 2.2e-16***	< 2.2e-16***	***E2***	< 2.2e-16***	< 2.2e-16***

*Abbreviations: HP01 = haphpipe_assemble_01 (de novo assembly), HP02 = haphpipe_assemble_02 (reference-based assembly), GDN = Geneious de novo assembly, GRB = Geneious reference-based assembly, pol = polymerase, combination of PRRT and int, PPRT = protease and reverse transcriptase, int = integrase, PDIST = genetic p-distance , APDIST = adjusted genetic p-distance, *** indicates p < 0.001.*

Table S3. Wilcoxon signed-rank comparisons of the genetic p-distance from the true sequence between the initial and final consensus sequences constructed in the HAPHPIPE pipelines for the simulation dataset.

	HP01				HP02			
**Sub B**	**pseudomedian**	**CI low**	**CI high**	**p value**	**pseudomedian**	**CI low**	**CI high**	**p value**
pol	-0.0005	NA[1]	NA[1]	1	0.0004	0.0004	0.0004	0.0004***
PRRT	-0.0002	NA[1]	NA[1]	1	0.0006	0.0006	0.0006	0.0006***
int	-0.0012	NA[1]	NA[1]	1	NA[1]	NA[1]	NA[1]	NA[1]
gp120	0.0007	NA[1]	NA[1]	1	0.0067	0.00514	0.0080	9.42E-14***
**Non-B**	**pseudomedian**	**CI low**	**CI high**	**p value**	**pseudomedian**	**CI low**	**CI high**	**p value**
pol	-0.0008	-0.0010	-0.0008	2.5E-09***	0.0008	0.0006	0.0012	0.0059**
PRRT	-0.0009	-0.0010	-0.0008	3.5E-09***	0.0013	0.0009	0.0018	0.0056**
int	-0.0012	-0.0017	-0.0011	1.2E-06***	NA[1]	NA[1]	NA[1]	NA[1]
gp120	-0.0007	-0.0009	-0.0006	4.7E-06***	0.0071	0.0045	0.0127	4.0E-06***

*[1] No confidence intervals were constructed because too many differences were zero. A positive value indicates that the refined sequences are more genetically similar to the reference sequence (HXB2), while a negative value indicates that the refined sequences are less genetically similar to the reference sequence (HXB2). Abbreviations: Non-B: non-subtype B sequences, Sub B: subtype B sequences, HP01 = haphpipe_assemble_01 (de novo assembly), HP02 = haphpipe_assemble_02 (reference-based assembly), GDN = Geneious de novo assembly, GRB = Geneious reference-based assembly, pol = polymerase, combination of PRRT and int, PRRT = protease and reverse transcriptase, int = integrase, CI: confidence interval, *** indicates p < 0.001, ** indicates p < 0.01.*

Table S4. Kruskal-Wallis multiple comparisons (Dunn test) of the genetic p-distance of the pipeline consensus sequence from the true sequence for the simulation dataset. P-values are reported (Holm adjustment).

		**Subtype B Simulation Data**					**Non-Subtype B Simulation Data**				
**Gene**	**Pipeline**	**HP01**	**HP02**	**GDN**	**GRB**	**HyDRA**	**HP01**	**HP02**	**GDN**	**GRB**	**HyDRA**
pol	**HP01**		0.1193	1.2E-25***	3.5E-27***	3.0E-73***		0.0075**	5.6E-13***	5.4E-19***	1.2E-40***
	**HP02**	0.1193		8.8E-18***	5.2E-19***	4.1E-59***	0.0075		1.9E-05***	2.7E-09***	2.1E-25***
	**GDN**	1.2E-25***	8.8E-18***		0.7396	1.4E-13***	5.6E-13***	1.9E-05***		0.0967	7.2E-09***
	**GRB**	3.5E-27***	5.2E-19***	0.7396		1.3E-12***	5.4E-19***	2.7E-09***	0.0967		3.4E-05***
	**HyDRA**	3.0E-73***	4.1E-59***	1.4E-13***	1.3E-12***		1.2E-40***	2.1E-25***	7.2E-09***	3.4E-05***	
PRRT	**HP01**		0.1074	4.5E-26	2.8E-27***	5.4E-74***		6.8E-05***	1.8E-39***	5.2E-16***	1.8E-39***
	**HP02**	0.1074		6.1E-18***	6.7E-19***	1.9E-59***	6.8E-05***		0.0763	0.0001***	1.7E-18***
	**GDN**	4.5E-26***	6.1E-18***		0.7916	1.4E-13***	1.8E-39***	0.0763		0.0474*	2.6E-11***
	**GRB**	2.8E-27***	6.7E-19***	0.7916		7.6E-13***	5.2E-16***	0.0001***	0.0474*		3.5E-06***
	**HyDRA**	5.4E-74***	1.9E-59***	1.4E-13***	7.6E-13***		1.8E-39***	1.7E-18***	2.6E-11***	3.5E-06***	
int	**HP01**		1	8.9E-23***	1.1E-23***	9.7E-69***		0.5034	2.1E-12***	1.2E-13***	6.0E-36***
	**HP02**	1		1.4E-21***	2.0E-22***	2.1E-66***	0.5034		3.9E-09***	3.7E-10***	6.4E-30***
	**GDN**	8.9E-23***	1.4E-21***		0.8292	9.0E-14***	2.1E-12***	3.9E-09***		0.6952	2.6E-07***
	**GRB**	1.1E-23***	2.0E-22***	0.8292		3.5E-13***	1.2E-13***	3.7E-10***	0.6952		1.6E-06***
	**HyDRA**	9.7E-69***	2.1E-66***	9.0E-14***	3.5E-13***		6.0E-36***	6.4E-30***	2.6E-07***	1.6E-06***	
gp120	**HP01**		1.2E-33***	0.7817	3.4E-33***	NA		1.4E-12***	0.4949	1.9E-17***	NA
	**HP02**	1.2E-33***		2.2E-29***	0.9207	NA	1.4E-12***		9.4E-15***	0.2973	NA
	**GDN**	0.7817	2.2E-29***		5.1E-29***	NA	0.4949	9.4E-15***		4.6E-20***	NA
	**GRB**	3.4E-33***	0.9207	5.1E-29***		NA	1.9E-17***	0.2973	4.6E-20***		NA

Abbreviations: HP01 = haphpipe_assemble_01 (de novo assembly), HP02 = haphpipe_assemble_02 (reference-based assembly), GDN = Geneious de novo assembly, GRB = Geneious reference-based assembly, pol = polymerase, combination of PRRT and int, PRRT = protease and reverse transcriptase, int = integrase, *** indicates $p < 0.001$, ** indicates $p < 0.01$, * indicates $p < 0.05$.

Table S5. Wilcoxon signed-rank comparisons of the genetic p-distance from HXB2, the HIV reference sequence, between the initial and final consensus sequences constructed in the HAPHPIPE pipelines for the simulation dataset.

	**HP01**				**HP02**			
**Sub B**	**pseudomedian**	**CI low**	**CI high**	**p value**	**pseudomedian**	**CI low**	**CI high**	**p value**
pol	-0.0004	NA[1]	NA[1]	1	-0.0004	-0.0004	-0.0004	0.0003***
PRRT	-0.0012	NA[1]	NA[1]	1	-0.0006	-0.0006	-0.0006	0.0006***
int	0.0012	NA[1]	NA[1]	1	NA[1]	NA[1]	NA[1]	NA[1]
gp120	0.0005	NA[1]	NA[1]	1	-0.0054	-0.0063	-0.0046	< 2.2e-16***
**Non-B**	**pseudomedian**	**CI low**	**CI high**	**p value**	**pseudomedian**	**CI low**	**CI high**	**p value**
pol	-0.0009	-0.0010	-0.0008	1.8E-09***	-0.0004	-0.0006	-0.0004	0.0016**
PRRT	-0.0009	-0.0010	-0.0008	4.6E-09***	-0.0006	-0.0009	-0.0005	0.0025**
int	-0.0012	-0.0017	-0.0012	2.1E-07***	-0.0023	NA[1]	NA[1]	1
gp120	-0.0006	-0.0007	-0.0005	5.9E-05***	-0.0015	-0.0019	-0.0012	2.9E-08***

*[1] No confidence intervals were constructed because too many differences were zero. A positive value indicates that the refined sequences are more genetically similar to the reference sequence (HXB2), while a negative value indicates that the refined sequences are less genetically similar to the reference sequence (HXB2). Abbreviations: Non-B: non-subtype B sequences, Sub B: subtype B sequences, HP01 = haphpipe_assemble_01 (de novo assembly), HP02 = haphpipe_assemble_02 (reference-based assembly), GDN = Geneious de novo assembly, GRB = Geneious reference-based assembly, pol = polymerase, combination of PRRT and int, PRRT = protease and reverse transcriptase, int = integrase, CI: confidence interval, *** indicates p < 0.001, ** indicates p < 0.01.*

Table S6. Kruskal-Wallis multiple comparisons (Dunn test) of the genetic p-distance of the pipeline consensus sequence from HXB2, the HIV reference sequence, for the simulation dataset. P-values are reported (Holm adjustment).

		**Subtype B Simulation Data**					**Non-Subtype B Simulation Data**				
**Gene**	**Pipeline**	**HP01**	**HP02**	**GDN**	**GRB**	**HyDRA**	**HP01**	**HP02**	**GDN**	**GRB**	**HyDRA**
pol	**HP01**		1	2.4E-08***	1.4E-08***	2.8E-38***		0.8790	0.0013**	0.0002***	1.2E-17***
	**HP02**	1		6.4E-08***	4.3E-08***	6.6E-37***	0.8790		0.0120*	0.0029**	7.9E-15***
	**GDN**	2.4E-08***	6.4E-08***		0.9069	3.1E-12***	0.0013**	0.0120*		0.6142	1.9E-06***
	**GRB**	1.4E-08***	4.3E-08***	0.9069		6.3E-12***	0.0002***	0.0029**	0.6142		2.2E-05***
	**HyDRA**	2.8E-38***	6.6E-37***	3.1E-12***	6.3E-12***		1.2E-17***	7.9E-15***	1.9E-06***	2.2E-05***	
PRRT	**HP01**		1	0.0001***	7.9E-05***	6.8E-24***		0.6221	0.0409*	0.0091**	4.7E-13***
	**HP02**	1		0.0004***	0.0003***	4.0E-22***	0.6221		0.3085	0.1239	6.0E-10***
	**GDN**	0.0001***	0.0004***		0.8995	1.0E-08***	0.0409*	0.3085		0.5987	7.8E-06***
	**GRB**	7.9E-05***	0.0003***	0.8995		1.9E-08***	0.0091**	0.1239	0.5987		8.7E-05***
	**HyDRA**	6.8E-24***	4.0E-22***	1.0E-08***	1.9E-08***		4.7E-13***	6.0E-10***	7.8E-06***	8.7E-05***	
int	**HP01**		0.9621	6.2E-12***	4.9E-12***	7.9E-49***		1	0.0002***	0.0001***	3.2E-19***
	**HP02**	0.9621		5.9E-12***	4.2E-12***	4.3E-49***	1		0.0005***	0.0003***	5.2E-18***
	**GDN**	6.2E-12***	5.9E-12***		1	4.8E-14***	0.0002***	0.0005***		0.8497	2.3E-06***
	**GRB**	4.9E-12***	4.2E-12***	1		9.4E-14***	0.0001***	0.0003***	0.8497		5.5E-06***
	**HyDRA**	7.9E-49***	4.3E-49***	4.8E-14***	9.4E-14***		3.2E-19***	5.2E-18***	2.3E-06***	5.5E-06***	
gp120	**HP01**		2.1E-07***	0.3860	0.0124*	NA		2.7E-11***	0.2534	4.2E-13***	NA
	**HP02**	2.1E-07***		1.3E-09***	0.0179*	NA	2.7E-11***		3.4E-16***	0.5408	NA
	**GDN**	0.3860	1.3E-09***		0.0013**	NA	0.2534	3.4E-16***		1.9E-18***	NA
	**GRB**	0.0124*	0.0179*	0.0013**		NA	4.2E-13***	0.5408	1.9E-18***		NA

Abbreviations: HP01 = haphpipe_assemble_01 (de novo assembly), HP02 = haphpipe_assemble_02 (reference-based assembly), GDN = Geneious de novo assembly, GRB = Geneious reference-based assembly, pol = polymerase, combination of PRRT and int, PRRT = protease and reverse transcriptase, int = integrase, *** indicates $p < 0.001$, ** indicates $p < 0.01$, * indicates $p < 0.05$.

Table S7. Kruskal-Wallis multiple comparisons (Dunn test) of the genetic p-distance of the pipeline consensus sequence from HXB2, the HIV reference sequence, for the HIV empirical dataset. Adjusted p-values are reported (Holm adjustment).

Gene	Pipeline	HP01	HP02	HP01 haps	HP02 haps	GDN	GRB	Hydra
PRRT	**HP01**		3.3E-15***	0.3874	3.5E-11***	1.3E-06***	1.1E-13***	6.3E-06***
	**HP02**	3.3E-15***		2.7E-32***	0.0084**	0.0329*	1	0.0133*
	**HP01 haps**	0.3874	2.7E-32***		2.9E-40***	5.9E-16***	2.0E-29***	1.3E-14***
	**HP02 haps**	3.5E-11***	0.0084**	2.9E-40***		0.7660	0.0407*	1
	**GDN**	1.3E-06***	0.0329*	5.9E-16***	0.7660		0.0836	1
	**GRB**	1.1E-13***	1	2.0E-29***	0.0407*	0.0836		0.0401*
	**HYDRA**	6.3E-06***	0.0133*	1.3E-14***	1	1	0.0401*	
int	**HP01**		1.6E-13***	1	8.6E-17***	4.2E-06***	8.3E-13***	8.4E-10***
	**HP02**	1.6E-13***		1.8E-26***	1	0.0906	0.8189	1
	**HP01 haps**	1	1.8E-26***		8.0E-54***	7.0E-13***	4.2E-25***	1.2E-19***
	**HP02 haps**	8.6E-17***	1	8.0E-54***		0.2353	1	1
	**GDN**	4.2E-06***	0.0906	7.0E-13***	0.2353		0.1581	1
	**GRB**	8.3E-13***	0.8189	4.2E-25***	1	0.1581		1
	**HYDRA**	8.4E-10***	1	1.2E-19***	1	1	1	
gp120	**HP01**		2.0E-14***	0.0570	2.1E-10***	0.0016**	2.4E-06***	NA
	**HP02**	2.0E-14***		2.1E-37***	0.0420*	0.0001***	0.0286*	NA
	**HP01 haps**	0.0570	2.1E-37		1.8E-39***	2.0E-12***	2.2E-19***	NA
	**HP02 haps**	2.1E-10***	0.0420*	1.8E-39***		0.0546	0.4317	NA
	**GDN**	0.0016**	0.0001***	2.0E-12***	0.0546		0.2876	NA
	**GRB**	2.4E-06***	0.0286*	2.2E-19***	0.4317	0.2876		NA

*Abbreviations: HP01 = haphpipe_assemble_01 (de novo assembly), HP02 = haphpipe_assemble_02 (reference-based assembly), GDN = Geneious de novo assembly, GRB = Geneious reference-based assembly, haps = haplotypes, pol = polymerase, combination of PRRT and int, PRRT = protease and reverse transcriptase, int = integrase, *** indicates p < 0.001, ** indicates p < 0.01, * indicates p < 0.05.*

Table S8. Kruskal-Wallis multiple comparisons (Dunn test) of the genetic p-distance of the pipeline consensus sequence from H77, the HCV reference sequence, for the HCV empirical dataset. Adjusted p-values are reported (Holm adjustment).

Gene	Pipeline	HP01	HP02	HP01 haps	HP02 haps	GDN	GRB	Hydra
core	**HP01**		4.3E-09***	0.7323	8.3E-12***	0.0395*	0.0134*	
	**HP02**	4.3E-09***		6.5E-12***	1	3.5E-17***	0.0056**	4.3E-09***
	**HP01 haps**	0.7323	6.5E-12***		7.8E-20***	0.0058**	0.0066**	0.7323
	**HP02 haps**	8.3E-12***	1	7.8E-20***		3.2E-23***	0.0027**	8.3E-12***
	**GDN**	0.0395*	3.5E-17***	0.0058**	3.2E-23***		5.5E-07***	0.0395*
	**GRB**	0.0134*	0.0056**	0.0066**	0.0027**	5.5E-07***		0.0134*
E1	**HP01**		2.1E-07***	0.9376	2.4E-07***	0.5735	4.7E-06***	
	**HP02**	2.1E-07***		6.0E-10***	1	1.1E-11***	1	2.1E-07***
	**HP01 haps**	0.9376	6.0E-10***		3.4E-11***	0.4746	4.7E-08***	0.9376
	**HP02 haps**	2.4E-07***	1	3.4E-11***		2.3E-12***	1	2.4E-07***
	**GDN**	0.5735	1.1E-11***	0.4746	2.3E-12***		6.4E-10***	0.5735
	**GRB**	4.7E-06***	1	4.7E-08***	1	6.4E-10***		4.7E-06***
E2	**HP01**		0.0001***	1	1.2E-06***	0.1492	3.2E-05***	
	**HP02**	0.0001***		6.0E-06***	0.8905	9.2E-10***	1	0.0001***
	**HP01 haps**	1	6.0E-06***		1.8E-10***	0.0245*	1.3E-06***	1
	**HP02 haps**	1.2E-06***	0.8905	1.8E-10***		4.7E-14***	1	1.2E-06***
	**GDN**	0.1492	9.2E-10***	0.0245*	4.7E-14***		1.7E-10***	0.1492
	**GRB**	3.2E-05***	1	1.3E-06***	1	1.7E-10***		3.2E-05***

*Abbreviations: HP01 = haphpipe_assemble_01 (de novo assembly), HP02 = haphpipe_assemble_02 (reference-based assembly), GDN = Geneious de novo assembly, GRB = Geneious reference-based assembly, haps = haplotypes, *** indicates p < 0.001, ** indicates p < 0.01, * indicates p < 0.05.*

Table S9. Wilcoxon signed-rank comparisons of the genetic p-distance from the reference sequence between the initial and final consensus sequences constructed in the HAPHPIPE pipelines for the empirical HIV and HCV datasets. The reference sequences for HIV and HCV were HXB2 and H77, respectively.

	HP01				HP02			
**Empirical HIV dataset**								
	**pseudomedian**	**CI low**	**CI high**	**p value**	**pseudomedian**	**CI low**	**CI high**	**p value**
PRRT	0.0022	0.0017	0.0035	1.5E-06***	NA[1]	NA[1]	NA[1]	NA[1]
Int	0.0021	0.0016	0.0031	2.2E-05***	-0.0012	NA[1]	NA[1]	1
gp120	0.0026	0.0020	0.0038	2.6E-07***	-0.0005	-0.0005	-0.0005	0.1736
**Empirical HCV dataset**								
	**pseudomedian**	**CI low**	**CI high**	**p value**	**pseudomedian**	**CI low**	**CI high**	**p value**
core	0.0083	0.0021	0.0125	0.0012**	0.0101	0.0025	0.1027	0.0412*
E1	0.0086	0.0054	0.0121	0.0001***	-0.0026	-0.0069	-0.0017	0.0579
E2	0.0079	0.0048	0.0144	0.0001***	-0.0027	-0.0041	-0.0009	0.0002***

*[1] No confidence intervals were constructed because too many differences were zero. A positive value indicates that the refined sequences are more genetically similar to the reference sequence, while a negative value indicates that the refined sequences are less genetically similar to the reference sequence. The reference sequence for the empirical HIV and HCV datasets were HXB2 and H77, respectively. Abbreviations: HP01 = haphpipe_assemble_01 (de novo assembly), HP02 = haphpipe_assemble_02 (reference-based assembly), GDN = Geneious de novo assembly, GRB = Geneious reference-based assembly, PRRT = protease and reverse transcriptase, int = integrase, CI: confidence interval, *** indicates p < 0.001, ** indicates p < 0.01.*

Table S10. Genetic p-distance to the reference sequence across the empirical SARS-CoV-2 dataset.

	Average	STDEV
**HP01 Inital**	0.0033	0.0035
**HP01 Final**	0.0035	0.0034
**HP02 Inital**	0.0019	0.0015
**HP02 Final**	0.0021	0.0019
**GDN**	0.0741	0.0407
**GRB**	0.0047	0.0011

Abbreviations: HP01 = haphpipe_assemble_01 (de novo assembly), HP02 = haphpipe_assemble_02 (reference-based assembly), GDN = Geneious de novo assembly, GRB = Geneious reference-based assembly, STDEV = standard deviation.

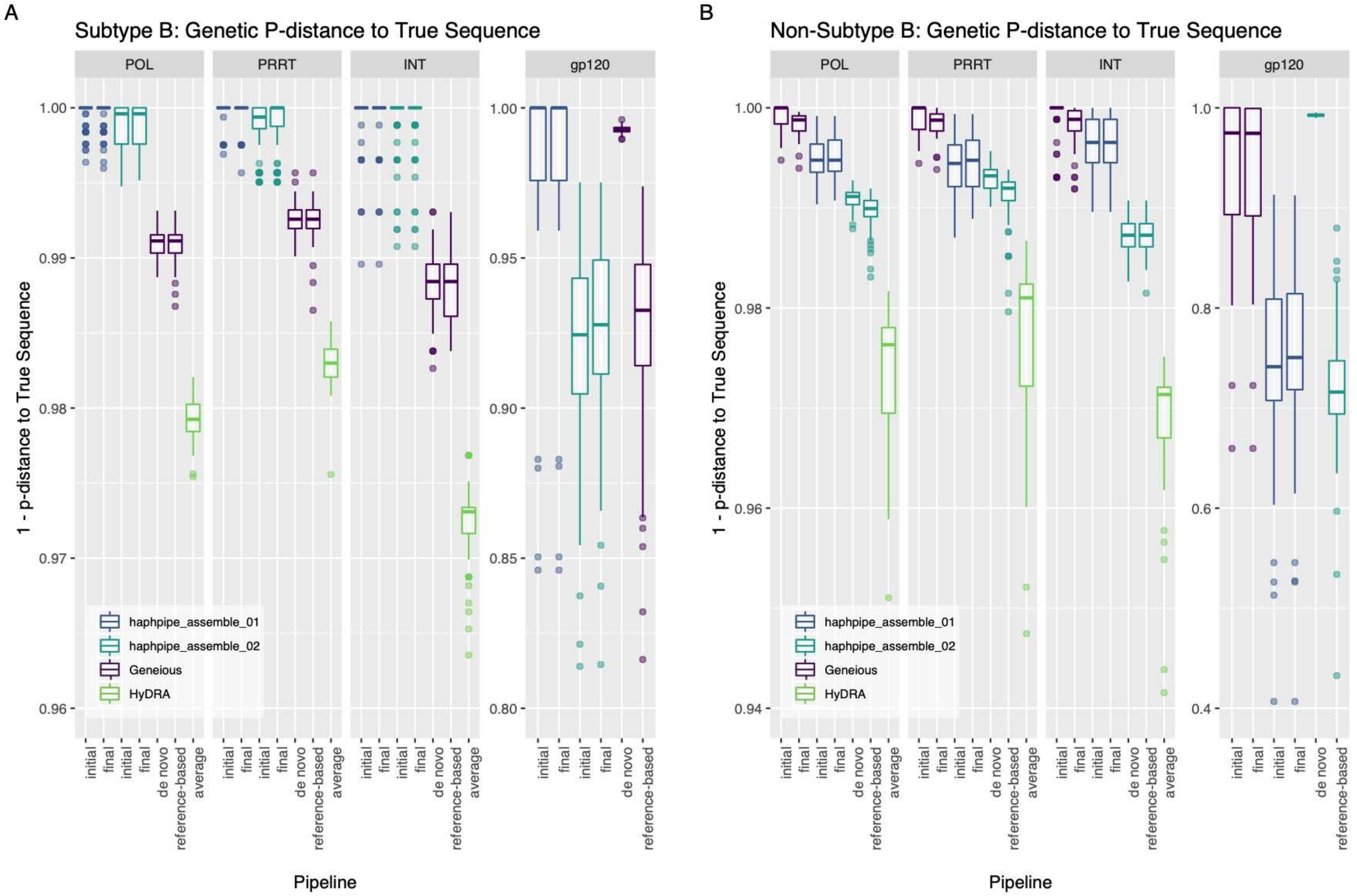
A
Subtype B: Genetic P-distance to True Sequence
POL
PRRT
INT
gp120
1 - p-distance to True Sequence
haphpipe_assemble_01
haphpipe_assemble_02
Geneious
HyDRA
initial
final
de novo
reference-based
average
Pipeline
B
Non-Subtype B: Genetic P-distance to True Sequence
POL
PRRT
INT
gp120
1 - p-distance to True Sequence
haphpipe_assemble_01
haphpipe_assemble_02
Geneious
HyDRA
initial
final
de novo
reference-based
average
Pipeline

Figure S1. Genetic p-distance (displayed as a difference from 1) between consensus sequence and true sequence for all pipelines for the simulated HIV **A**) subtype B dataset and **B**) non-subtype B dataset. A value closer to 1.00 indicates the consensus sequence is more genetically similar to the true sequence. The x-axis order from left to right for an individual panel: adjusted genetic p-distance between the true sequence and (i) the initial assembled sequence followed by (ii) the final assemble sequence for haphpipe_assemble_01 pipeline (de novo assembly); (iii) the initial assembled sequence followed by (iv) the final assemble sequence for haphpipe_assemble_02 pipeline (reference-based assembly); the final consensus sequence for the Geneious (v) de novo workflow and the (vi) reference-based workflow; and finally, the (vi) average between the final two sequences (one for each read file) for HyDRA. The three amplicons are shown, as well as a combination of *PRRT* and *int* amplicons into *pol*. There are no results for HyDRA in the *gp120* gene because HyDRA only analyzes the *pol* gene.

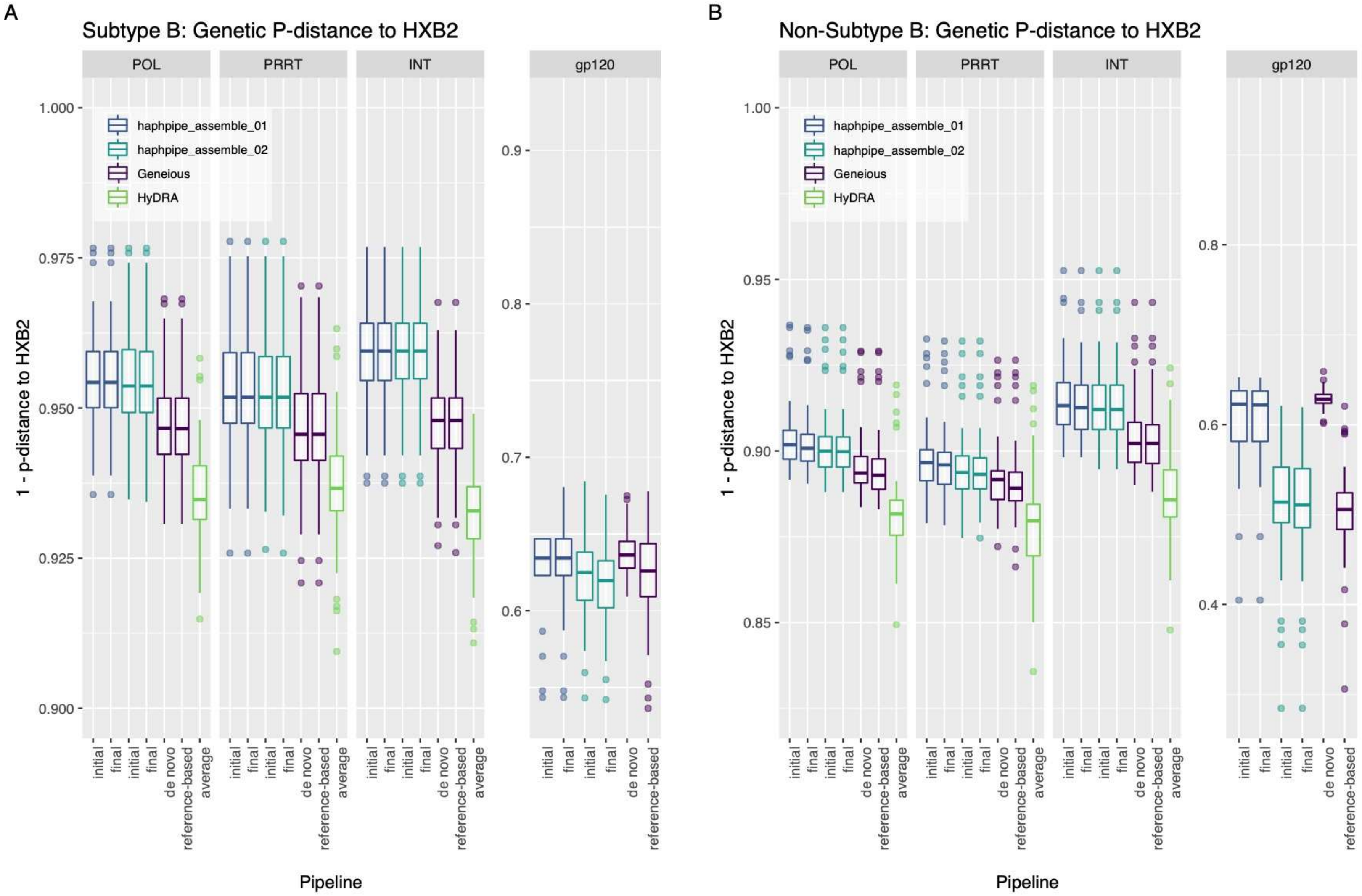
A
Subtype B: Genetic P-distance to HXB2
B
Non-Subtype B: Genetic P-distance to HXB2
POL
PRRT
INT
gp120
haphpipe_assemble_01
haphpipe_assemble_02
Geneious
HyDRA
1 - p-distance to HXB2
initial
final
de novo
reference-based
average
Pipeline

Figure S2. Genetic p-distance (displayed as a difference from 1) between consensus sequence and HXB2, the reference sequence for HIV, for all pipelines for the simulated HIV **A**) subtype B dataset and **B**) non-subtype B dataset. Ambiguous nucleotides were accounted for by giving fractional weight in alignment. A value closer to 1.00 indicates that the consensus sequence is more genetically similar to the reference sequence. The x-axis order from left to right for an individual panel: adjusted genetic p-distance between the reference sequence and (i) the initial assembled sequence followed by (ii) the final assemble sequence for haphpipe_assemble_01 pipeline (de novo assembly); (iii) the initial assembled sequence followed by (iv) the final assemble sequence for haphpipe_assemble_02 pipeline (reference-based assembly); the final consensus sequence for the Geneious (v) de novo workflow and the (vi) reference-based workflow; and finally, the (vi) average between the final two sequences (one for each read file) for HyDRA. The three amplicons are shown, as well as a combination of *PRRT* and *int* amplicons into *pol*. There are no results for HyDRA in the *gp120* gene because HyDRA only analyzes the *pol* gene.

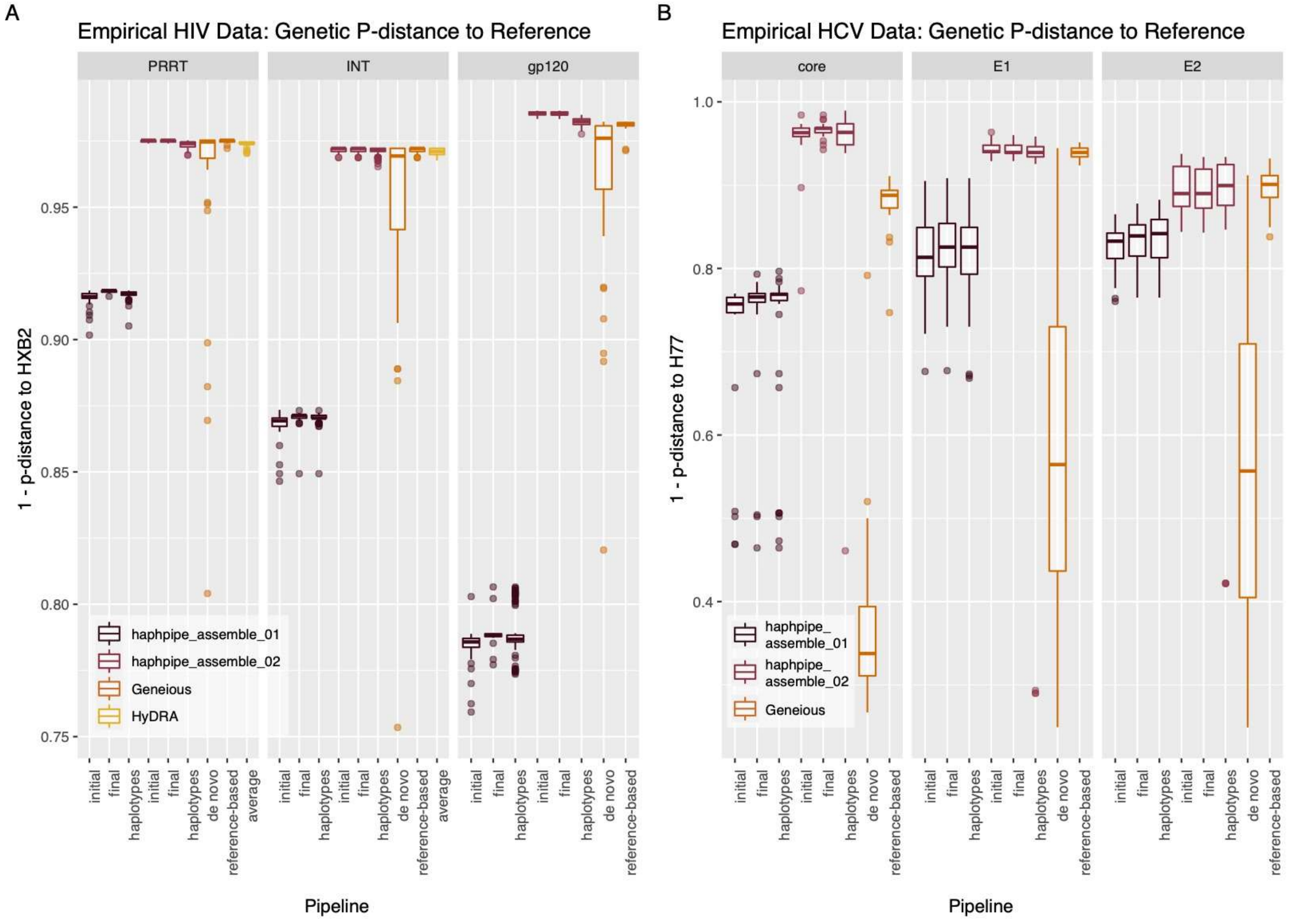
A
Empirical HIV Data: Genetic P-distance to Reference
PRRT
INT
gp120
1 - p-distance to HXB2
0.95
0.90
0.85
0.80
0.75
haphpipe_assemble_01
haphpipe_assemble_02
Geneious
HyDRA
initial
final
haplotypes
de novo
reference-based
average
Pipeline
B
Empirical HCV Data: Genetic P-distance to Reference
core
E1
E2
1 - p-distance to H77
1.0
0.8
0.6
0.4
haphpipe_assemble_01
haphpipe_assemble_02
Geneious
Pipeline

Figure S3. Genetic p-distance (displayed as a difference from 1) between consensus sequence and HXB2, the reference sequence for HIV, for all pipelines for the empirical **A)** HIV dataset and **B**) HCV dataset. Ambiguous nucleotides were accounted for by giving fractional weight in alignment. A value closer to 1.00 indicates that the consensus sequence is more genetically similar to the reference sequence. The y-axes are different for each HIV and HCV, with HCV showing greater variance between samples. The x-axis order from left to right for an individual panel: adjusted genetic p-distance between the reference sequence and (i) the initial assembled sequence, (ii) the final assemble sequence and (iii) the reconstructed haplotypes for haphpipe_assemble_01 pipeline (de novo assembly); (iv) the initial assembled sequence, (v) the final assemble sequence, and (vi) the reconstructed haplotypes for haphpipe_assemble_02 pipeline (reference-based assembly); the final consensus sequence for the Geneious (vii) de novo workflow and (vi) reference-based workflow; and finally, the (viii) average between the final two sequences (one for each read file) for HyDRA. The three amplicons are shown for both empirical datasets (HIV: *PRRT*, *int*, *gp120* and HCV: *core*, *E1*, *E2*). There are no results for HyDRA in the *gp120* gene for HIV or for any HCV genes because HyDRA only analyzes the *pol* gene region of HIV.
